# A clinical study of serum lipid disturbance in Chinese patients with sudden deafness

**DOI:** 10.1186/1476-511X-12-95

**Published:** 2013-07-03

**Authors:** Tingwen Weng, Erin E Devine, Hongming Xu, Zhisong Yan, Pin Dong

**Affiliations:** 1 Department of Cardiology, Huadong Hospital, Fudan University, Shanghai 200040, People’s Republic of China; 2 Department of Surgery-Division of Otolaryngology- Head and Neck Surgery, University of Wisconsin School of Medicine and Public Health, Madison, WI 53706, USA; 3 Department of Otolaryngology- Head and Neck Surgery, Shanghai First People’s Hospital, Shanghai Jiao Tong University, 100th Haining Road, Shanghai 200080, People’s Republic of China; 4 Tongzhou Hospital, Department of Otolaryngology, Nantong, Jiangsu 226300, People’s Republic of China; 5 Department of Otolaryngology- Head and Neck Surgery, Shanghai First People’s Hospital, Shanghai Jiao Tong University, Shanghai 200080, People’s Republic of China

**Keywords:** Sudden Hearing Loss, Cholesterol, Low Density Lipoprotein Cholesterol, Triglycerides

## Abstract

**Background:**

Pathological levels of blood lipids could be one of the causes of sudden sensorineural hearing loss (SSNHL). The objective of this manuscript is therefore to evaluate the relationship between blood lipid content and sudden sensorineural hearing loss (SSNHL).

**Methods:**

The correlation between serum lipid parameters, including total cholesterol (TC), triglycerides, high-density lipoprotein cholesterol (HDL-C), low density lipoprotein cholesterol (LDL-C), apolipoprotein AI (apo AI), apolipoprotein B (apo B), and lipoprotein A (Lp(a)), and the onset of SSNHL was analyzed from a data set of 250 patients and an age, gender and weight matched control group of 250 subjects.

**Results:**

The result of single factor logistic regression shows that TC (p<0.01), LDL-C (p<0.01), and apo B (p=0.03) of SSNHL group were significantly higher than those of the control group. The odds ratio of TC, LDL, and apo B are higher than 1, while the confidence intervals of the odds rations do not include 1. No significant difference was found with the prevalence of hypertension (P=0.818), diabetes (P=0.869) and smoking habits (P=0.653) between SSNHL group and control group.

**Conclusion:**

Total cholesterol, low density lipoprotein cholesterol and apolipoprotein B concentrations may be important factors in the pathogenesis of sudden sensorineural hearing loss, and should be assessed during the investigation of patients with this condition.

## Background

Sudden sensorineural hearing loss (SSNHL) is generally accepted by clinicians as a rapid hearing loss of >30 dB in at least three contiguous frequencies over the course of under 3 days [1]. Prevalence rates have been reported as being from 5–20 per 100,000 people in the United States, but this is likely to be an underestimate, since many who recover quickly never seek medical attention [2]. The prevalence in Asia has not been found significantly different, with an estimate of 13 per 100,000 based on a survey of Japanese hospitals [3]. SSNHL is reportedly associated with many vascular and coagulation diseases [2]. However, there is still debate as to the effect of blood serum proteins and lipids.

Inner ear function is greatly influenced by ischemia since the blood supply of the inner ear is dependent on the end arteriole [4]. Dyslipidemia is one of the most significant cardiovascular risk factors [5], which is also defined as a cardiovascular risk factor in the Chinese population [6], and evidence has shown that there is relationship between SSNHL and dyslipidemia [7-9]. Lipid-lowering therapies have been correlated with better hearing improvement in SSNHL patients than a standard treatment control group [10]. However, there are not enough large-scale clinical studies to support the correlation between SSNHL and dyslipidemia [11-13], which limits the therapeutic development of SSNHL. This study, a retrospective study into the serum lipid data of 250 SSNHL patients, evaluates the relationship between blood lipid and sudden sensorineural hearing loss.

## Methods

### Patients and controls

250 SSNHL patients (113 females and 137 males, with a mean age of 56.41 years ranging from 15–84 years), admitted between January 1, 2007 and December 30, 2012 in a single clinical hospital in Nantong China, were included in this study. The study was approved by and performed in accordance with the ethical standards of the hospital ethics committee. Patients included in the study visited the hospital for the first time within 7 days after the onset of SSNHL. Standard laboratory tests and audiological diagnostic procedures were performed in all subjects. We included patients with an average hearing loss of more than 30 dB for speech frequencies, and excluded patients if they had other diseases that may cause hearing loss, such as otitis media, ototoxic drugs, and noise trauma. We also excluded patients with malignant disease psychiatric illness, dementia, hepatitis B or C, or severe systemic diseases. A normal gender, age, and weight matched group without hearing disease, comprised of 250 patients scheduled for nasal endoscopic surgery (e.g. functional endoscopic sinus surgery), was used for comparison. The exclusion criteria were the same as for the SSNHL group.

### Test procedures

All hearing was evaluated by pure tone audiometry and conducted in the same audiological laboratory using a Madsen clinical audiometer (MADSEN midimate622 Diagnostic Audiometers). Inner ear CT scans or MRI scans were performed in all the patients, and no inner ear structural abnormality or tumors were found. The evaluation was performed after hospital admission. Laboratory parameters were also evaluated. Blood samples were drawn from patients and control subjects after overnight fast in the morning between 6 and 7 am. These parameters included: total cholesterol (TC) concentration; low density lipoprotein cholesterol (LDL-C); high density lipoprotein cholesterol (HDL-C); triglycerides (TG); apolipoprotein AI (Apo A1); apolipoprotein B (Apo B); and lipoprotein A (Lp(a)). Any other standard tests indicated by the physician were also performed. The following ranges were considered normal: TC of 3.6-6.5 mmol/L; LDL-C of 2.5-3.5 mmol/L; HDL-C:1.1-1.7 mmol/L; TG of <2.0 mmol/L; apo AI: 1.2-1.76 g/L; apo B: 0.6-1.14 g/L; and Lp(a): 0-300 mg/L.

### Statistical analysis

Sigmaplot12 was used to perform statistical analyses. All continuous variables were presented as mean±standard deviation (x±SD). Single factor logistic regression was carried out to analyze the correlation between serum lipid parameters and the onset of SSNHL. It is assumed the dependent variable of SSNHL group is “Y=1”, the dependent variable of control group is “Y=0”, and the lipid parameters are independent variables. The chi-square test was employed to compare the prevalence of hypertension, diabetes and smoking habits between SSNHL group and control group.

## Results

**Figure 1:**
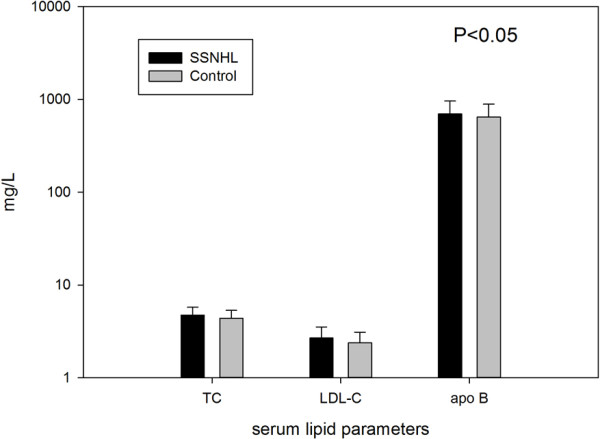
Comparison of TC (total cholesterol) and LDL-C (low density lipoprotein cholesterol) between SSNHL (sudden sensorineural hearing loss) group and control group.

**Figure 2:**
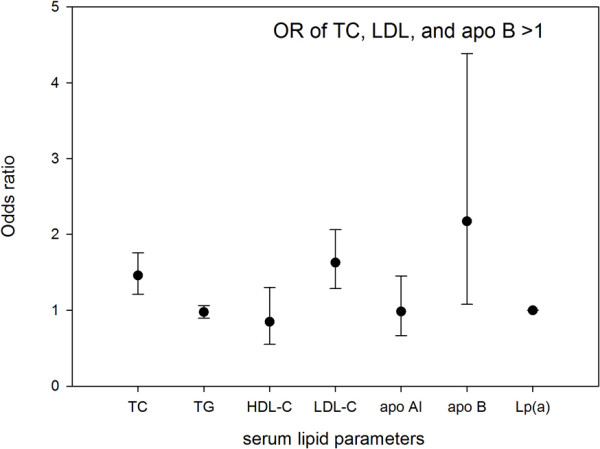
Odds ratio and confidence interval of lipid parameters (TC- total cholesterol; TG- triglycerides; HDL-C- high density lipoprotein cholesterol; LDL-C- low density lipoprotein cholesterol; Apo A1- apolipoprotein A1; Apo B- apolipoprotein B; Lp(a)- lipoprotein A).

The average of each parameter for both SSNHL and control conditions can be seen in Table 1. The means of serum lipid data of both groups were in the normal range. Table 2 gives the result of single factor logistic regression and Figure 1 shows that TC (p<0.01), LDL-C (p<0.01), and apo B (p=0.03) of SSNHL group were significantly higher than those of the control group. Figure 2 shows the odds ratio of TC, LDL, and apo B are higher than “1”, while the confidence intervals of the odds rations do not include “1”. No significant difference was found with the prevalence of hypertension (P=0.818), diabetes (P=0.869) and smoking habits (P=0.653) between SSNHL group and control group.

**Table 1:** The average of serum lipid data of both groups is in the normal range

	**Normal range**	**SSNHL group(n=250)**	**Control group(n=250)**
TC(mmol/L)	3.6-6.5	4.738±1.021	4.378±0.937
TG(mmol/L)	0-1.71	1.322±0.967	1.449±3.249
HDL-C(mmol/L)	1.1-1.7	1.432±0.401	1.459±0.420
LDL-C(mmol/L)	2.5-3.5	2.679±0.856	2.380±0.723
apoAI(g/L)	1.2-1.76	1.422±0.448	1.425±0.460
apoB(g/L)	0.6-1.14	0.695±0.266	0.645±0.242
Lp(a)(mg/L)	0-300	194.008±183.906	221.368±260.077

**Table 2:** Single factor logistic regression shows TC, LDL-C, and apo B of SSNHL group are significantly higher than those of control group, which indicate TC, LDL-C, and apo B correlate with SSNHL

**Variants**	**Regression coefficient b**	**Standard error**	**Wald statistic**	**P value**	**OR(95% CI)**
TC	0.378	0.095	15.747	<0.01	1.459(1.211-1.758)
TG	-0.0243	0.043	0.0317	0.574	0.976(0.897-1.062)
HDL-C	-0.166	0.219	0.576	0.448	0.847(0.552-1.300)
LDL-C	0.488	0.120	16.451	<0.01	1.628(1.287-2.061)
apo AI	-0.0167	0.198	0.007	0.933	0.983(0.667-1.450)
apo B	0.776	0.358	4.699	0.030	2.173(1.077-4.385)
Lp(a)	-0.0005	0.0004	1.820	0.177	0.999(0.999-1.000)

The confidence intervals of odds ratio don’t include “1”.

## Discussion

The etiology of SSNHL is not well understood at present. There are different theories about its etiology and risk factors of SSNHL are a topic of debate across the literature [14]. Some clinical research indicates that risk factors of SSNHL overlap those of cardiovascular disease, such as hyperfibrinogenemia and smoking [14,15]. As far as the correlation between dyslipidemia and SSNHL, opinions vary. A clinical study of 86 subjects in China showed that the levels of total cholesterol (TC), triglyceride (TG), and lipoprotein A (Lp(a)) were significantly higher in patients with SSNHL than in control subjects [15]. This study indicated plasma viscosity, ratio viscosity of whole blood, reduced viscosity of whole blood, high and low shear relative viscosity of whole blood, index of red blood cells transmutation, and fibrinogen level in the plasma of patients with SSNHL were also significantly elevated in comparison with those in control subjects. In a retrospective study, Oreskovic reported that patients with SSNHL had significantly higher plasma concentrations of cholesterol and low-density lipoprotein cholesterol, compared with controls [7]. However, their test group was on average 15 years older than the control group, which may have influenced the results. In addition, it is suggested that fibrinogen/LDL apheresis may improve cochlear blood flow by acutely decreasing plasma cholesterol and fibrinogen [11,12]. However, it is hard to say whether the therapeutic effect is gained from lowing fibrinogen or LDL. There are also some negative reports of the relationship between dyslipidemia and SSNHL [14]. In order to add to the clinical evidence of this problem and to avoid selection bias, we carried out this retrospective study with the data of SSNHL patients in a medium-sized clinical hospital of Nantong, China, which is a moderately developed medium-sized city, instead of major medical institutions.

It is generally accepted that serum lipid level is correlated with age and body mass index (BMI) [16,17]. Therefore, we matched our disordered group with normal subjects who have the same age, gender and similar weight to avoid these confounding factors. It is also believed hypertension, diabetes and smoking habits could impair vascular tone and integrity in the vascular bed of the ears, thus could promoting SSNHL [4]. We compared the prevalence of hypertension, diabetes and smoking habits between the SSNHL and control groups, and no significant difference was found. The average of serum lipid data of both groups is in the normal range. Similar results have been reported in previous studies [7,9].

Approximately 40% of LDL particles are enriched with cholesterol, so serum LDL-C levels are related to TC level. A batch of clinical and epidemiological studies demonstrate a continuous, graded relationship of serum LDL level to long-term risk of coronary heart disease and cardiovascular disease (CVD) [5,18,19]. LDL enters the vascular wall through endothelial cells and is detained in subendothelial tissue and converted to the oxidized form(Ox-LDL). Macrophages are engulfed by Ox-LDLs and converted into xanthoma cells. The proliferation and coalescing of xanthoma cell then turns into the lipid core of an atherosclerotic plaque. Therefore, serum level of TC and LDL-C are closely related with thrombotic diseases and some scholars believe cardiovascular risk also has a significant impact on the onset of SSNHL [20,21]. Meanwhile, high cholesterol not only affects the inner ear blood supply [21], but can also affect the activities of outer hair cells by damaging their structure [22]. Apo B is an elementary composition of LDL-C, which is essential for the binding of LDL particles to the LDL receptor, allowing cells to internalize LDL and thus absorb cholesterol. A considerable amount of data exists to indicate that apo B is a strong predictor of CVD risk [23]. Our study has shown that TC, LDL-C, and apo B of SSNHL group are significantly higher than those of the control group, which correlates well with the mechanism of these serum lipid parameters leading to CVD. On the other hand, HDL-C and apo AI are considered to be protective factors of CVD [24]. In this study, SSNHL patients also showed lower HDL-C and apo AI level than controls but this trend was not statistically significant.

As far as mechanism is concerned, there are a lot of studies indicating that dyslipidemia can cause SSNHL by affecting the inner ear blood supply. An animal experiment shows endothelial dysfunction in cerebral arterioles were significantly increased in apoE(-/-) mice on the high-fat diet >6 months compared with the control group [25]. Another animal experiment found that blood flow in the cochleas was reduced significantly in hypertensive rats exposed to an atherogenic diet compared to that of normotensive or hypertensive control animals [26]. It is generally accepted that Nitric oxide, a potent vasodilator plays an extremely important role in the blood supply of inner ear [27]. LDL cholesterol can impair NO release, influencing the blood supply of inner ear, while dyslipidemia [28]. On the other hand, dyslipidemia can cause SSNHL by damaging the inner ear structure. After the administration of a hyperlipid diet for 3 months, histochemical study of the guinea pigs’ inner ear revealed variations in lipid metabolism and partial disorders of the outer hair cells while electron microscopic observations showed vacuolar and parenchymal protrusions on the surfaces of the striavascularis and Corti’s organ [29]. It has also been reported that cholesterol has different distributions among outer hair cell membranes, and when water-soluble cholesterol is incorporated into the cells, the outer hair cell lateral wall stiffness parameter increases, which impairs the activity of outer hair cell [22].

One of the major limitations of this study is the observational design. In order to avoid bias, we tried to collect as much patient data as possible. Since dyslipidemia may cause SSNHL by affecting the inner ear blood supply with the forming of atherosclerosis plaque, which is also the pathogeny of cardiovascular disease, we did not exclude the patients with CVD. However, no other vascular studies were done in all patients.

## Conclusion

This retrospective study of 250 SSNHL patients indicates total cholesterol and low density lipoprotein cholesterol concentrations may be important factors in the pathogenesis of sudden sensorineural hearing loss, and should be assessed during the investigation of patients with this condition.

## Abbreviations

SSNHL: Sudden sensorineural hearing loss; TC: Total cholesterol; TG: Triglycerides; HDL: High density lipoprotein; HDL-C: High density lipoprotein cholesterol; LDL: Low density lipoprotein; LDL-C: Low density lipoprotein cholesterol; Apo A1: Apolipoprotein A1; Apo B: Apolipoprotein B; Lp(a): Lipoprotein A; dB: Decibels; CT: Computed tomography; MRI: Magnatic resonance imaging; Ox-LDL: Oxidized low density lipoprotein; CVD: Cardiovascular disease.

## Competing interests

The authors have no financial or competing interests to disclose.

## Authors’ contributions

HX participated in study design, data collection. TW participated in manuscript preparation and statistical analysis. ED was involved in data analysis and manuscript preparation. ZY was involved in data collection and patient test procedures. PD was involved in interpretation. All authors read and approved the final manuscript.

## Authors’ information

HX received his MD from Jiaotong University and is an otolaryngologist for Shanghai First People’s Hospital, Shanghai Jiao Tong University in Shanghai, China. TW received her MD from Fudan University and is a cardiologist Fudan University affiliated Huadong Hospital in Shanghai, China. ED received her MS in Biomedical Engineering from the University of Wisconsin-Madison and works as an Assistant Researcher for the Division of Otolaryngology, Department of Surgery, University of Wisconsin School of Medicine and Public Health. ZY received his MD from Nantong University and is an otolaryngologist for Tongzhou Hospital, in Nantong, Jiangsu, China. PD received his MD and PHD from Shandong University and is an otolaryngologist for Shanghai First People’s Hospital, Shanghai Jiao Tong University in Shanghai, China.
